# Does immunosenescence drive organismal ageing via inflammageing?

**DOI:** 10.1186/s12979-021-00242-z

**Published:** 2021-07-06

**Authors:** Graham Pawelec

**Affiliations:** 1 Department of Immunology, University of Tübingen, Tübingen, Germany; 2 Health Sciences North Research Institute, Sudbury, Ontario Canada

For many years, a major question in ageing research has been whether organismal ageing processes affect all tissues similarly, or whether ageing of one organ system drives deficits in others, as originally proposed by Roy Walford in the context of autoimmunity a half-century ago [1]. Yousefzadeh et al. now report that engineering defective DNA repair exclusively in hematopoietic stem cells (HSCs) increases the accumulation of DNA damage in immune cells and results in their early senescence as reflected by higher expression of p16 and p21 [2]. They further show that there were many similarities between these Ercc1-deficient animals at younger ages and naturally aged mice at older ages. Moreover, strikingly, p16 and p21 expression was higher in many non-immune tissues of animals deficient for Ercc1 only in cells derived from HSCs, resulting in widespread tissue damage. These data provide strong support for a modern version of the “immunological theory of ageing” in that senescence limited only to the immune system in this model drives the ageing phenotype and occurrence of degenerative diseases of ageing to a striking degree. This important conclusion rests upon the absolute limitation of Ercc1-deficiency solely to HSCs. Here there may be some provisos to this model which are intrinsic to the use of tissue-specific transgenes to inactivate a gene of interest in a certain organ or tissue. First, there may be leakage of expression of the CRE-expressing transgene in other tissues, inactivating the gene of interest in (a fraction of) cells of that tissue as well, compromising the tissue-specificity of the gene inactivation technique. This was addressed by Yousefzadeh et al. excluding massive leakage but a minor loss of Ercc1 in other tissues is difficult to exclude with the techniques applied and may have been missed, especially in older animals. Second, in a small fraction of cells, inactivation of the floxed gene may not occur and these cells may outgrow the majority that has inactivated the gene of interest, if the wild-type cells have a growth advantage. Both phenomena may apply to tissue-specific Ercc1 mutants. Some other instances of “leakage” in a different context include increased levels of the oxidative DNA lesion 8-oxo-guanine in Ercc1-deficient immune cells, despite the fact that these lesions are repaired by BER, which is not affected by the Ercc1 repair defect. This may suggest “knock-on” effects to other DNA repair mechanisms. Conversely, DNA damage as manifested by cyclopurines was not affected by Ercc1-deficiency but this would normally be expected because these lesions are usually repaired by Ercc1. Nonetheless, these intriguing results make a major contribution to the question of whether immunosenescence drives ageing of other tissues in that even if there is some “leakage” the main effect seems clearly to be due to the activities of immune cells rather than senescence in general, as seen when Ercc1 is knocked out in the whole organism [3].

In the second part of the paper, the authors take this notion a step further, by showing that the premature organismal ageing phenotype of Ercc1-deficient mice could be prevented in several tissues by infusion of splenocytes from normal young mice. Reciprocally, transfer of splenocytes from either younger Ercc1-deficient animals or unmanipulated old animals induced senescence in several tissues of young mice [2]. These are remarkable data suggesting that deficits in only one DNA repair mechanism in only one stem cell compartment can result in immune senescence that is shown to impair not only both cellular and humoural immunity but also to indirectly affect the apparent ageing phenotypes of non-HSC-derived tissues, and moreover paralleling many degenerative changes seen in naturally ageing mice. These fascinating findings raise many questions, not least of which is in how far these findings in mice might also apply to humans. This is not susceptible to investigation because Ercc-1 mutations in humans are exceedingly rare, cause severe developmental dysfunction and are not limited to HSCs. Nonetheless, there is a long-standing school of thought that “inflammageing” links organismal and immune ageing via inflammatory mediators produced by senescent immune cells (but also many other senescent cell types and known as the “senescence-associated secretory phenotype”, or SASP) [4]. Yousefzadeh et al. now provide strong supporting evidence that it may indeed be senescent immune cells and not senescent cells in general that drive inflammageing. If so, a declining ability of immune cells to clear other senescent cells [5] as they themselves become senescent could constitute a vicious circle feedback. Hence, the “anti-ageing” effect of infusions of young splenocytes into Ercc1-deficient mice might be mediated by the capacity of the young cells to clear both senescent immune and nonimmune cells. This remains to be explored.

## Data Availability

Not applicable.
